# Upregulation of pERK and c-JUN by γ-tocotrienol and not α-tocopherol are essential to the differential effect on apoptosis in prostate cancer cells

**DOI:** 10.1186/s12885-020-06947-6

**Published:** 2020-05-15

**Authors:** Christine Moore, Victoria E. Palau, Rashid Mahboob, Janet Lightner, William Stone, Koyamangalath Krishnan

**Affiliations:** 1 Division of Hematology-Oncology Department of Internal Medicine, James H. Quillen College of Medicine, East Tennessee State University, Dogwood Avenue, Building 119, Johnson City, USA; 2 Department of Pharmaceutical Sciences, Bill Gatton College of Pharmacy, East Tennessee State University, Johnson City, TN 37614 USA; 3 Wellmont Hospitalists at Kingsport, Kingsport, TN 37660 USA; 4 Department of Pediatrics, James H. Quillen College of Medicine, East Tennessee State University, Johnson City, TN 37614 USA

**Keywords:** γ-Tocotrienol, α-Tocopherol, Prostate cancer, Prevention, Differential effect, Vitamin E isoforms

## Abstract

**Background:**

α-tocopherol (AT) and γ-tocotrienol (GT3) are vitamin E isoforms considered to have potential chemopreventive properties. AT has been widely studied in vitro and in clinical trials with mixed results. The latest clinical study (SELECT trial) tested AT in prostate cancer patients, determined that AT provided no benefit, and could promote cancer. Conversely, GT3 has shown antineoplastic properties in several in vitro studies, with no clinical studies published to date. GT3 causes apoptosis via upregulation of the JNK pathway; however, inhibition results in a partial block of cell death. We compared side by side the mechanistic differences in these cells in response to AT and GT3.

**Methods:**

The effects of GT3 and AT were studied on androgen sensitive LNCaP and androgen independent PC-3 prostate cancer cells. Their cytotoxic effects were analyzed via MTT and confirmed by metabolic assays measuring ATP. Cellular pathways were studied by immunoblot. Quantitative analysis and the determination of relationships between cell signaling events were analyzed for both agents tested. Non-cancerous prostate RWPE-1 cells were also included as a control.

**Results:**

The RAF/RAS/ERK pathway was significantly activated by GT3 in LNCaP and PC-3 cells but not by AT. This activation is essential for the apoptotic affect by GT3 as demonstrated the complete inhibition of apoptosis by MEK1 inhibitor U0126. Phospho-c-JUN was upregulated by GT3 but not AT. No changes were observed on AKT for either agent, and no release of cytochrome c into the cytoplasm was detected. Caspases 9 and 3 were efficiently activated by GT3 on both cell lines irrespective of androgen sensitivity, but not in cells dosed with AT. Cell viability of non-cancerous RWPE-1 cells was affected neither by GT3 nor AT.

**Conclusions:**

c-JUN is a recognized master regulator of apoptosis as shown previously in prostate cancer. However, the mechanism of action of GT3 in these cells also include a significant activation of ERK which is essential for the apoptotic effect of GT3. The activation of both, ERK and c-JUN, is required for apoptosis and may suggest a relevant step in ensuring circumvention of mechanisms of resistance related to the constitutive activation of MEK1.

## Background

Prostate cancer is the most common malignancy in males, accounting for 19% of new cancer cases in males and 29,430 deaths in 2018 [1]. Most forms are either curable using local interventions, or indolent requiring timely surveillance. However, some forms are aggressive and metastasize to bones and other organs, leading to early morbidity and mortality [2]**.** Epidemiological studies suggest that disease progression may be associated to environmental factors and lifestyle, with a specific emphasis on diet [3]. Thus, prostate cancer prevalence and behavior makes it an ideal target for chemoprevention, or the use of chemical agents to disrupt progression of the disease to invasive cancer [4]. Among potential antineoplastic agents, antioxidants are known to exert protective mechanisms on healthy cells that may prevent disease progression. Vitamin E is an antioxidant, abundant in certain nuts, whole grains, and vegetables. It is an essential lipid-soluble vitamin composed of eight isoforms, four tocopherols and four tocotrienols (α, β, δ, and γ for each of these). Among these isoforms, α-tocopherol (AT) has the highest bioavailability [5] and its properties have been evaluated in clinical studies. Indeed, AT was the earliest isoform to be considered as a potential agent in prostate cancer prevention in a male smokers study. However, AT was used as a synthetic all-racemic-alpha tocopheryl acetate (rac-ATA) form with very promising beneficial results [6] thus gaining the acceptance of this synthetic molecule. Rac-ATA was used recently in the Selenium, Vitamin E and Prostate Cancer Chemoprevention Trial (SELECT), with very different outcomes [7]; it was concluded that this synthetic form of AT alone or in combination with selenium has no chemo preventive properties, and in fact could promote prostate cancer. The SELECT trial was not the first clinical study that showed a lack of antineoplastic effects by AT, in fact it confirmed the results obtained in the Women Health Study, which used a natural source of AT in cancers of the lung, breast and colon [8]. Conversely, an extensive number of research studies in vitro and in animal models suggest that tocotrienols have important antineoplastic effects in cancers of the breast, liver, pancreas, and prostate, but no clinical studies have evaluated their potential therapeutic use. Among the tocotrienols, γ-tocotrienol (GT3) has been studied in cancers of the breast, pancreas and prostate [9], where it has shown that it inhibits cell proliferation and cell invasion via chemotherapy sensitization, and by suppressing EGFR, NF-κB, and Id proteins, and activating the JNK signaling pathway and caspases 9 and 3. While these studies suggest GT3 as a potential chemopreventive agent that may be useful as an adjunctive treatment option for some forms of prostate cancer, the role of the RAF/RAS/ERK MAPK pathway and the relationship with other signaling cascades has not been clearly established. Here we present the specific mechanistic differences between AT and GT3 in both androgen-sensitive and androgen-independent prostate cancer cells and its multipronged effect on crucial cell signaling pathways affecting proliferation and survival.

## Methods

### Cell lines and culture conditions

The androgen independent PC-3 (CRL-1435) prostate cancer cell line, derived from a bone metastasis of a grade IV human prostatic adenocarcinoma displaying epithelial morphology, the androgen sensitive LNCaP (CRL-1740) prostate cancer cells, derived from a left supraclavicular lymph node of human prostate carcinoma, and RWPE-1 primary prostate epithelial cells, were purchased from the American Type Culture Collection (Manassas, VA). These cells were maintained as recommended by the supplier. Briefly, the prostate cancer cells were grown in RPMI1640 (CRL-1169) supplemented with 10% FBS, and penicillin/streptomycin (Life Technologies, Grand Island, NY), and the primary prostate epithelial cells were grown in keratinocyte SFM medium supplemented with growth factors (Fisher Scientific, Waltham, MA). For cytotoxicity and metabolism activity assays, the cells were seeded on 96 well plates at a density of 5 × 103 cells/well. For immunoblotting experiments, the cells were seeded on 60 mm plates at a density of at 5 × 10^5^ cells/cm^2^.

### Reagents

GT3 (98% pure), was purchased from Cayman Chemical (Ann Arbor, MI). The concentration of GT3 in ethanol was determined using an HP-8542A diode array spectrophotometer with the following maximum wavelengths (λ_max_) and molar extinction coefficients (ε): GT3 λ_max_ 298 nm, ε = 4230. AT (97% pure) was purchased from Tama Biochemical (Tokyo, Japan), its concentration was determined in the same fashion as for GT3.

### MTT assay

LNCaP, PC-3, and RWPE-1 cells were dosed with GT3 or AT at concentrations of 10, 20, 40, 60, and 80 μM. After treatment, 3-(4, 5-methylthiazol-2-yl)-2, 5-diphenyl-tetrazolium bromide (MTT) was added and incubated for 3 h (Invitrogen, Carlsbad, CA). Formazan products were solubilized with acidified SDS overnight. Optical density was measured at 570 nm using a Spectramax Plus spectrophotometer (Molecular Devices, Sunnyvale, CA, USA). All experiments were done at least three times.

### Cell viability assay by measuring the presence of ATP

Cells on tissue culture 96 well plates were treated with either GT3 or AT in concentrations of 10, 20, 40, 60, and 80 μM; dissolution vehicle was ethanol. Following 6, 12 and 24 h of treatment, cells were allowed to come to room temperature before adding 100 μL/well of CellTiter Glo® reagent from Promega (Madison, WI, USA) that measures ATP via a luciferase reaction. Luminescence indicative of the presence of ATP was measured on a luminometer (Promega).

### Western blot analysis

LNCaP, PC-3, and RWPE-1 cells were treated with indicated concentrations of GT3, AT, or vehicle as a control for 6 or 12 h. Harvested cells were lysed with RIPA Buffer (150 mM NaCl, 1% sodium deoxycholate, 1% Triton, 0.1% SDS, 10 mM Tris, 100 μM sodium orthovanadate, 50 mM sodium fluoride) containing phosphatase and protease inhibitors (Sigma, St. Louis, MO). The protein concentrations of the cell lysates were determined by the BCA protein assay (Cytoskeleton, Denver, CO). The samples were run in SDS-PAGE and blotted onto nitrocellulose or PVDF membranes (Pall Life Sciences, Ann Arbor, MI). Blotted membranes were processed according to recommended protocols for each antibody against total and phosphorylated forms of ERK (9101 and 9102), c-JUN (9165 and 9164), BAD (5284), and mitochondrial marker COX IV, clone 3E11 (Cell Signaling, Danvers, MA, USA). Antibodies against AKT, clone SK703 (EMD Millipore, Billerica, MA), cytochrome C clone 7H8.2C12 (BD Biosciences, San Jose, CA), and actin from Sigma (St. Louis, MO). Caspase activation was analyzed with anti- cleaved caspase 3, and cleaved caspase 9 from Cell Signaling (clones 5A1E, D2D4), Abcam and R&D systems. The signal of primary monoclonal or polyclonal antibodies was detected using affinity–purified secondary antibodies with no cross-reactivity with other species, coupled to peroxidase (Pierce and Promega, Fitchburg, WI, USA) and analyzed by a chemiluminescent system. The intensities of the bands on x-ray film were estimated by digitizing the image using Image J, and were compared against a control.

### Statistical analysis

Data are represented as the mean ± SE. Comparisons were done relative to the control, and analyses were run by Student’s t test when comparing against a control, or ANOVA followed by Bonferroni test when comparing two treatment groups. *p* < 0.05, *p* < 0.01 and *p* < 0.001 indicate statistical significance. (GraphPad Prism 7, La Jolla, CA). The data are shown with error bars representing standard deviation.

## Results

**Fig. 1:**
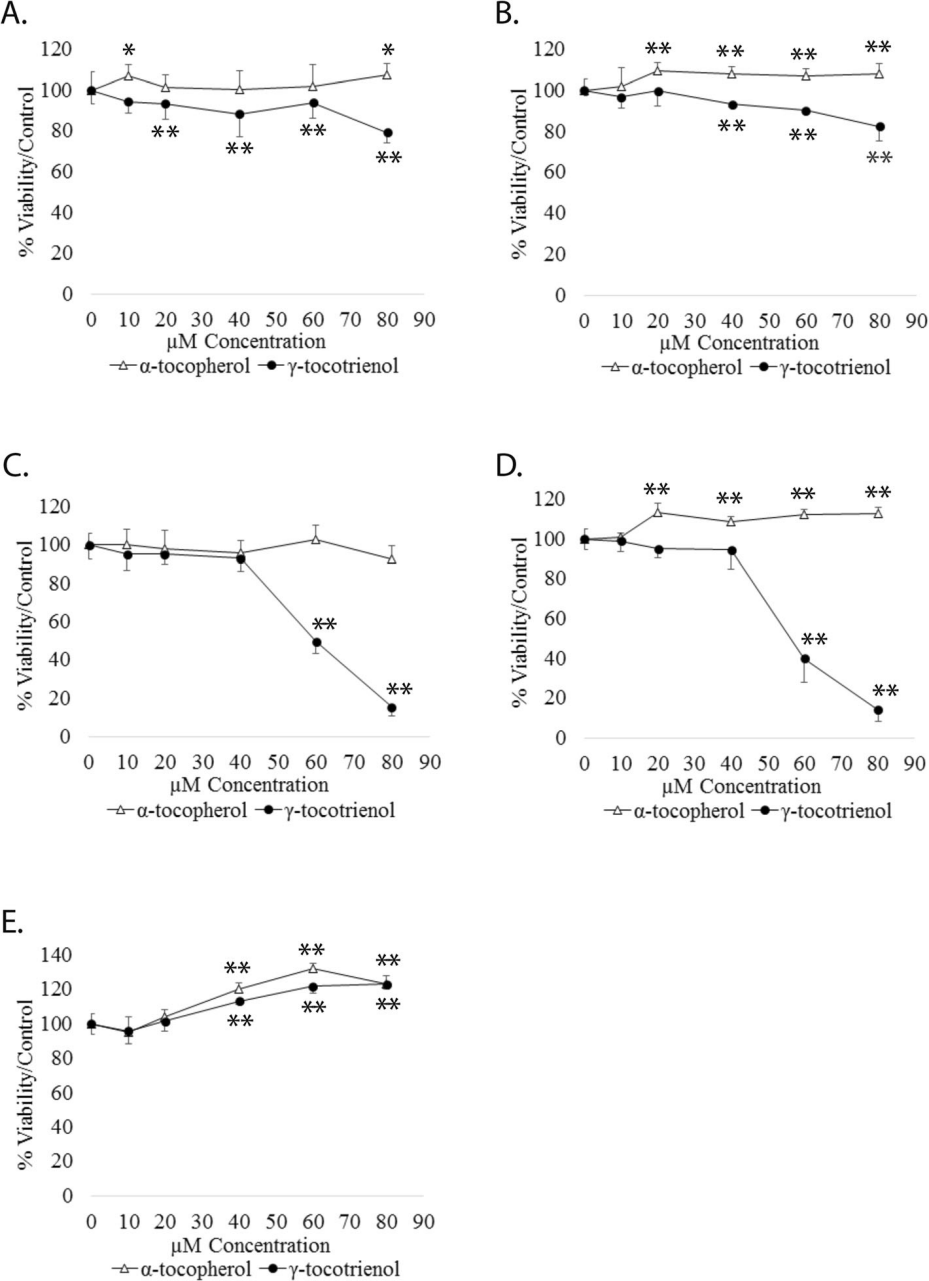
Effect of AT and GT3 on prostate cancer cells. **a** and **b**: LNCaP and PC-3 were treated with AT or GT3 at doses ranging from 10 to 80 μM. After 6 h of treatment, cell viability was determined via MTT. **c**, **d** and **e**: LNCaP, PC-3, and non-tumorigenic RWPE-1 cells underwent the same treatment as described above for 12 h. All graphs shown correspond to data obtained by analysis of metabolic activity from at least three independent experiments. For quantification spectrophotometric data were calculated as percentages of the value for the untreated cells (100%) ± standard deviations *n* = 3, *p < 0.05, **p < 0.01

*GT3 inhibits cell viability in a time and dose dependent manner in androgen-sensitive LNCaP and androgen-independent PC-3 prostate cancer cells, but not AT.* Recent findings indicate that AT may promote proliferation of prostate cancer cells [7]. Conversely, it has been reported that GT3 may cause apoptosis on prostate cancer cells [9]. To test whether these effects are sustained and time dependent LNCaP and PC-3 prostate cancer cells were dosed with either GT3 or AT at concentrations ranging from 5 to 80 μM. MTT and cell viability assays detecting the presence of ATP were run at 6 and 12 h after dosing. Both assays revealed similar trends; the results shown in Fig. 1 are of MTT data. At 6 h, LNCaP (Fig. 1a) and PC3 (Fig. 1b) cells dosed with AT show a constant trend towards sustaining cell viability and slight increase in proliferation at 80 μM. The effect of GT3 at lower concentrations is similar to that of AT. However, a downward trend is apparent at concentrations above 40 μM suggesting loss of cell viability and inhibition of metabolic activity. The MTT and metabolic activity assays at 12 h after dosing show that the effects observed at 6 h continue their trend, with a significantly larger difference in the effect of AT and GT3 on both cell lines at concentrations above 40 μM (Fig. 1c and d). Previous studies on prostate, have reported no inhibition of cell viability on normal cells. This observation is confirmed via MTT and metabolic activity assays on non-cancerous prostate cells RWPE-1 after dosing with AT or GT3 (Fig. 1e).

**Fig. 2:**
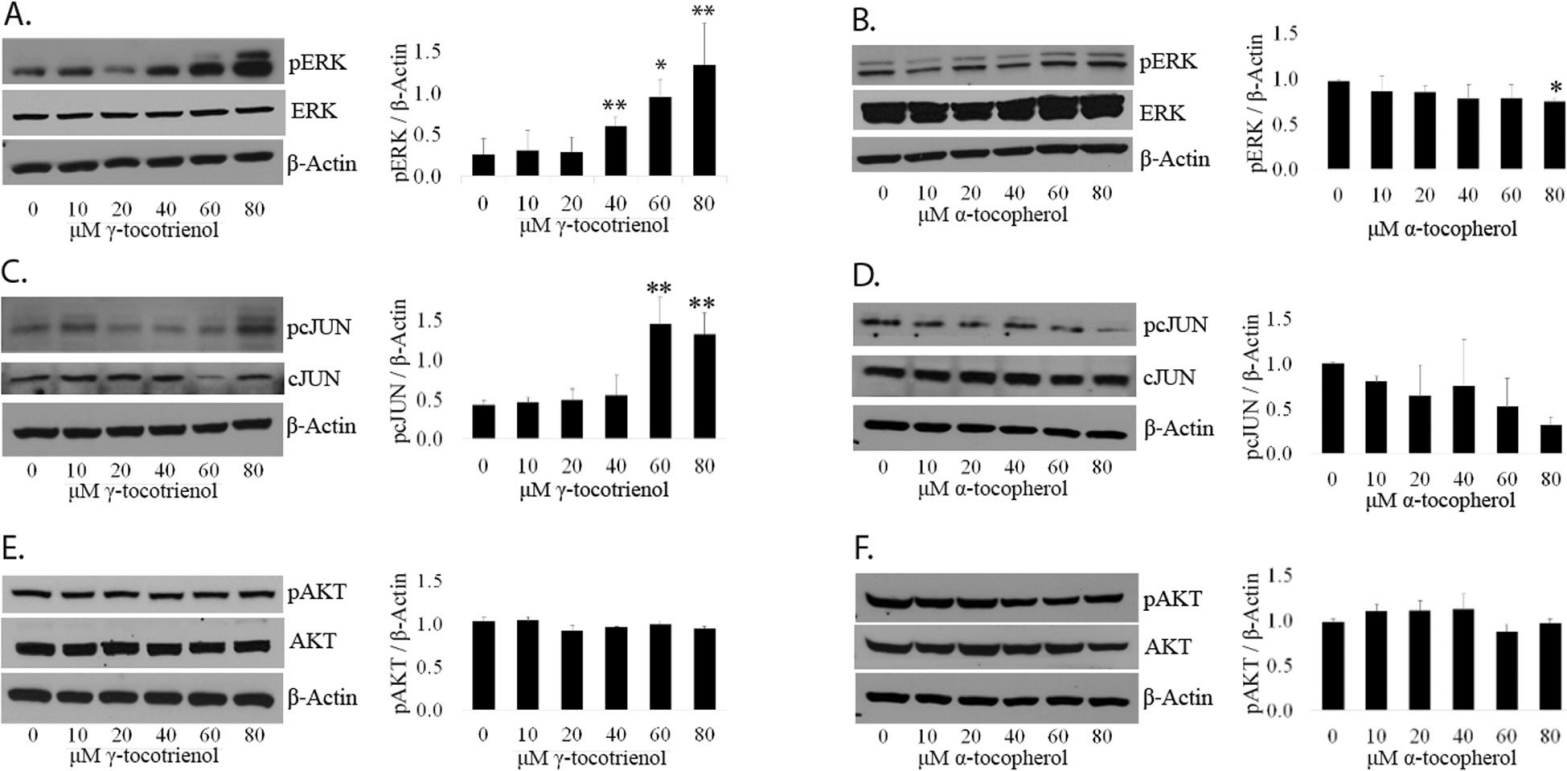
Involvement of the pERK, pc-JUN and pAKT in the response to treatment with GT3 or AT of LNCaP prostate cancer cells. **a**, **c**, and **e**: LNCaP cells were grown on 6-wellplates and treated with GT3 for 6 h at doses ranging from 10 to 80 μM. Immmunoblots from SDS total extracts were obtained using antibodies against total and phosphorylated forms of ERK (**a**) (**c**) c-JUN, and (**e**) AKT. **b**, **d**, and F: Similarly, LNCaP cells treated with AT for 6 h at the same dose range as described above. Immunoblots from total SDS extracts using antibodies against the total and phosphorylated forms of ERK (**b**), c-JUN (**d**) and AKT (**f**) are shown above. The membranes were reprobed for β-actin as a loading control. The results presented are representative of three separate experiments. The values are averages ± standard deviations from three independent experiments;*p < 0.05, **p < 0.01. Autoradiographs of the original blots are available in the Supplementary Section Figure S5.

**Fig. 3:**
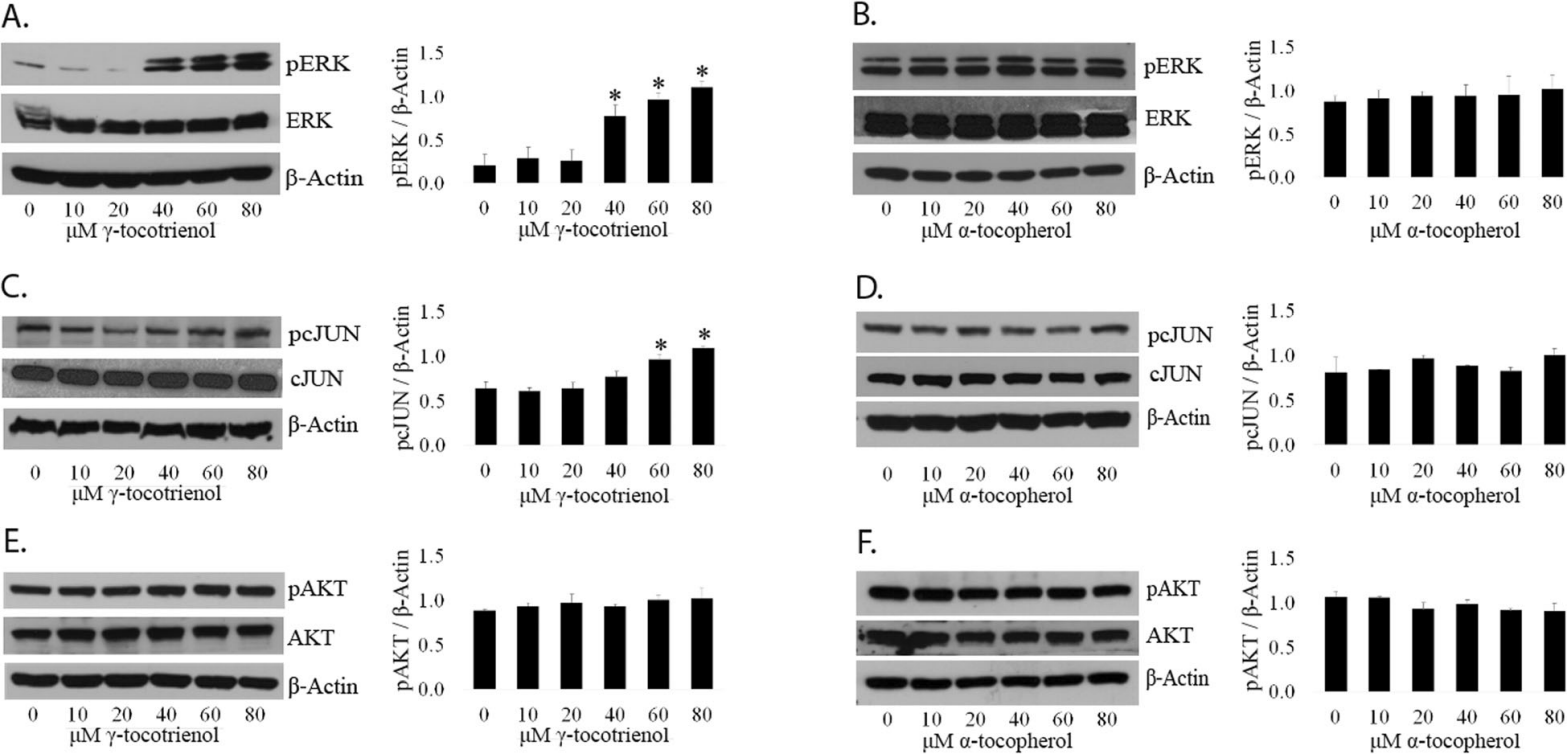
Involvement of the pERK, pc-JUN and pAKT in the response to treatment with GT3 or AT of PC3 prostate cancer cells. **a**, **c**, and **e**: PC3 cells were grown on 6-well plates and treated with GT3 in the same manner as described above. Immunoblots were obtained from SDS total extracts by using antibodies against the total and phosphorylated forms of ERK (**a**), c-JUN (**c**), and AKT (**e**). **b**, **d**, and **f**: Immunoblots of SDS total extracts obtained from PC3 cells treated with AT as described above, were obtained using antibodies against the phosphorylated and total forms of ERK (**b**) c-JUN (**d**), and AKT (**f**). Cells that were treated with neither GT3 nor AT, only with dissolution vehicle, were run as controls for each experiment. The membranes were reprobed for β-actin as a loading control. The results presented are representative of three separate experiments. The values are averages ± standard deviations from three independent experiments;*p < 0.05. Autoradiographs of the original blots are available in the Supplementary Section Figure S6.

*Activation of both, ERK and c-JUN are observed in LNCaP and PC3 cells after treatment with GT3 but not AT.* It has been reported that GT3 activates the c-JUN N-terminal kinase JNK signaling pathway in prostate cancer cells, with the subsequent phosphorylation of c-JUN [9]. In this study, it was shown that the addition of a specific JNK inhibitor was able to block partially the activity of the protein, suggesting a potential additional source of activation by GT3. Since ERK1/2 can phosphorylate c-JUN [10] we decided to probe the phosphorylation status of these proteins via immunoblot after dosing LNCaP and PC3 cells with GT3 or AT. Analysis of LNCaP cells show a 1.98 fold increase in the levels of the phosphorylated form of ERK after dosing with 80 μM of GT3 as compared to the control (Fig. 2a). Phospho-c-JUN follows a similar trend, and its levels increase 1.45 and 1.32 fold after dosing with 60 and 80 μM of GT3 (Fig. 2c). Conversely, in cells dosed with AT, the phosphorylated form of ERK is significantly lower (− 22.68%) at 80 μM, and a dramatic decrease (− 68%) for phospho-c-JUN (Fig. 2b and d). There is no change in the levels of ERK and cJUN as evidenced after normalization with β-actin (Figure S1, supplemental data). As in LNCaP cells, a similar trend is observed in PC3 cells. The expression levels of phospho-ERK show a significant fold increase of 3.71, 4.57, and 5.29 after dosing with GT3 at concentrations of 40, 60 and 80 μM respectively (Fig. 3a). The levels of the phosphorylated form of c-JUN show an appreciable fold increase of 1.50 and 1.69 at 60 and 80 μM, respectively. (Fig. 3c). In the case of AT, there is no significant change in these proteins after dosing (Fig. 3b and d). There is no change in the levels of ERK and cJUN as a result of treatment of PC3 cells with GT3 or AT, as evidenced after normalization with β-actin (Figure S2, supplemental data).

**Fig. 4:**
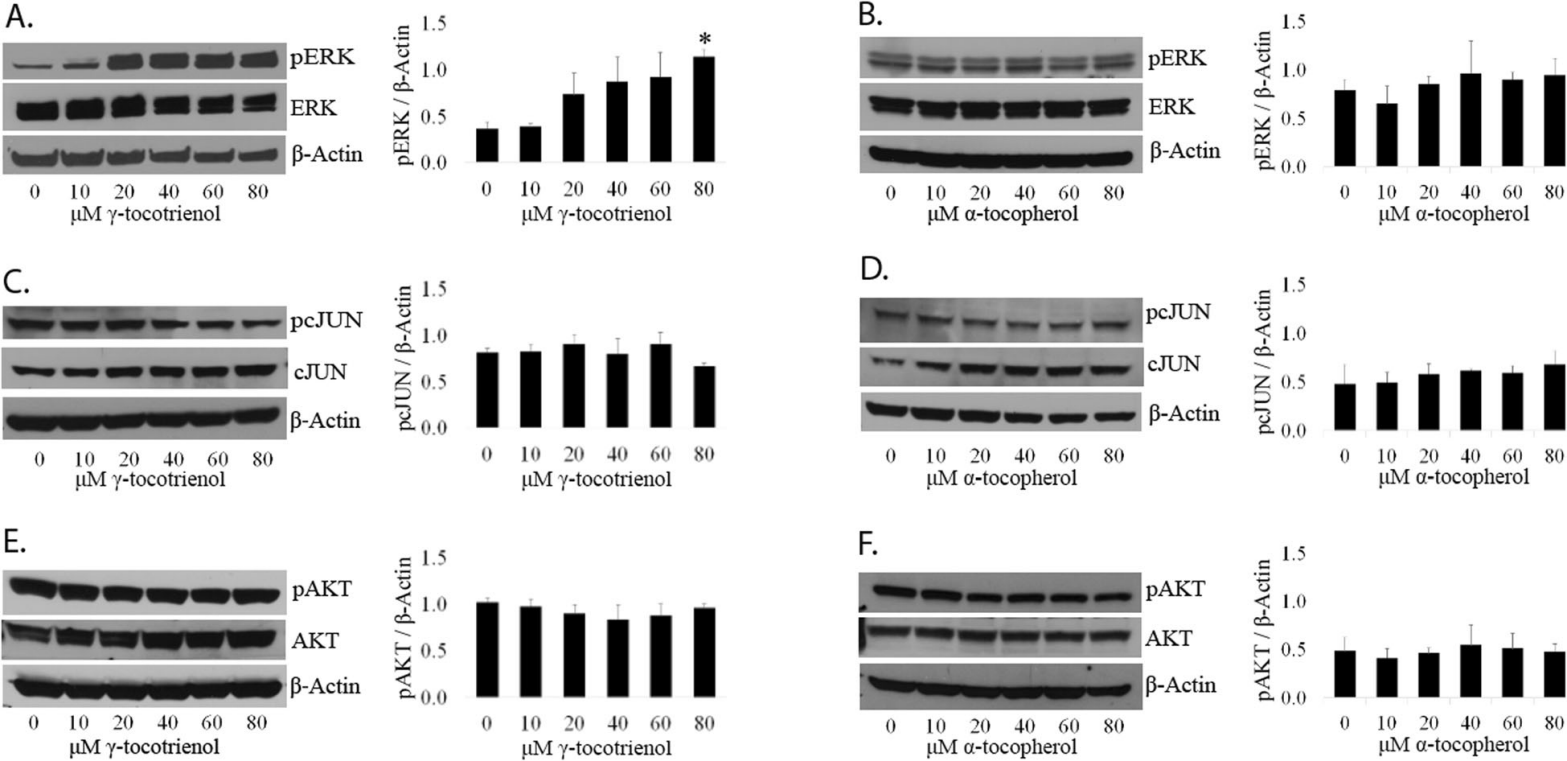
GT3 and AT have a similar effect on non-tumerigenic prostate cells. **a** and **b**: RWPE-1 cells grown on 6-wellplates and treated with (**a**) GT3 or (**b**) AT for 6 h at doses ranging from 10 to 80 μM were analyzed by immunoblot using antibodies against the total and phosphorylated forms of ERK. **c** and **d**: RWPE-1 cells were treated as described above, and analyzed via immunoblot. SDS total extracts of (**c**) GT3 or (**d**) AT treated cells were probed using antibodies against total and activated c-JUN. **e** and **f**: The expression levels of total and activated AKT were analyzed via immunoblot in RWPE-1 cells treated with (**e**) GT3 or (**f**) AT. The membranes were reprobed for β-actin as al loading control. The values are averages ± standard deviations from at least three independent experiments; *p < 0.05. Autoradiographs of the original blots are available in the Supplementary Section Figure S7

*Neither GT3 nor AT has an effect on the expression levels of the activated form of AKT on LNCaP and PC3 cells.* To test whether GT3 or AT have an effect on cell survival via the PI3K pathway, we determined the expression levels of the phosphorylated form of AKT at serine 473, a survival marker. LNCaP (Fig. 2e and f) and PC3 cells (Fig. 3e and f) dosed with GT3 or AT showed no significant changes on the expression levels of phospho-AKT as compared to the control, within the concentration range tested. There is no change in the levels of AKT in LNCaP and PC3 cells treated with GT3 or AT as evidenced after normalization with β-actin (Figures S1, and S2 supplemental data). In the case of non-cancerous prostate cells RWPE-1, there is no significant change on the activated forms of ERK (Fig. 4b), c-JUN (Fig. 4c and d), or AKT (Fig. 4e and f) after treatment with GT3 or AT, except for phospho-ERK after treatment with GT3 80 μM, which shows a fold increase of 3.08 (Fig. 4a).

There is also no change in ERK, c-JUN, and AKT after treatment of these cells with GT3 or AT (Figure S3, supplemental data).

**Fig. 5:**
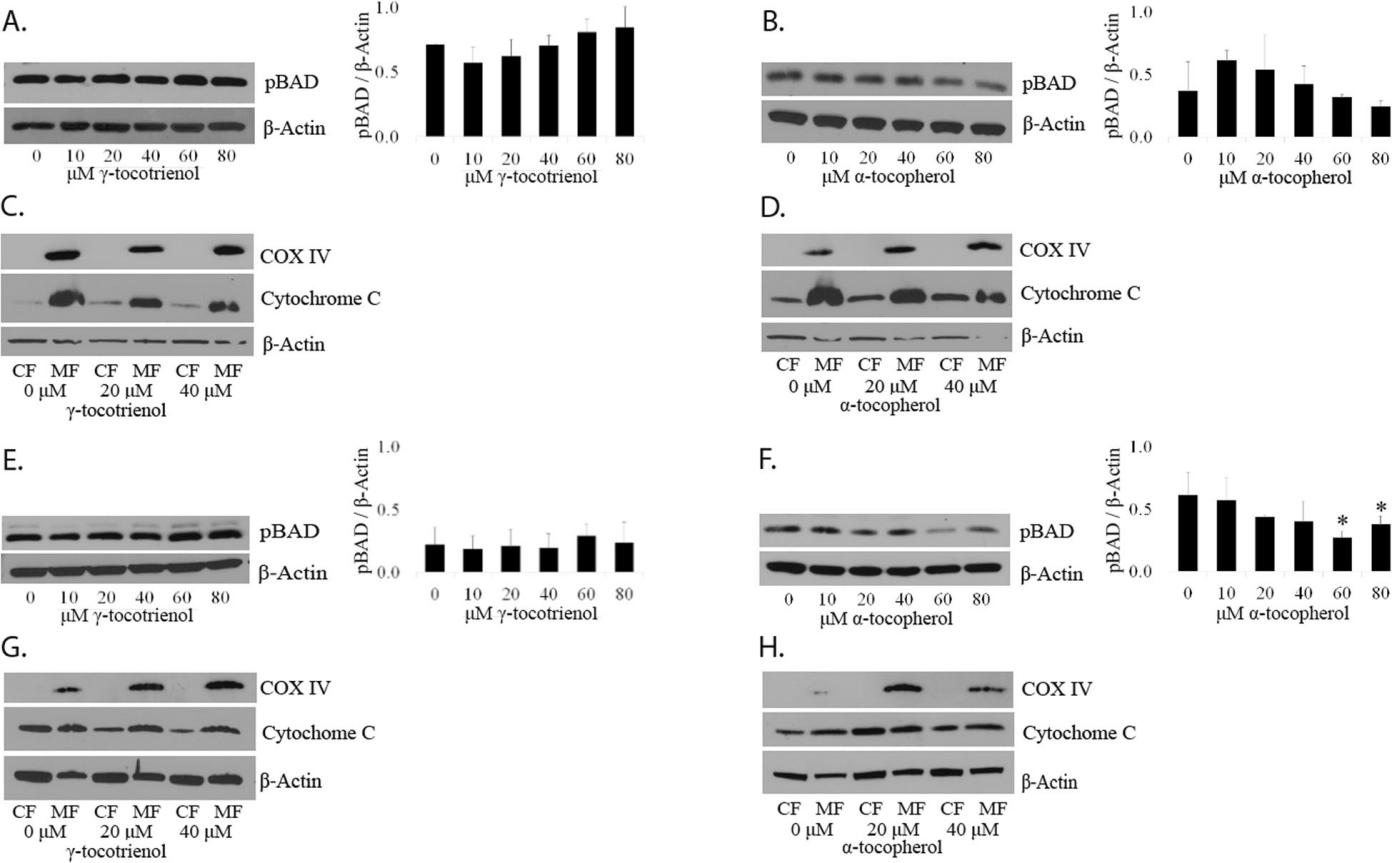
Effect on mitochondrial function of GT3 and AT on prostate cancer cells. **a** and **b**: LNCaP cells grown on 6-well plates and treated with (**a**) GT3 or (**b**) AT for 6 h at doses ranging from 10 to 80 μM were analyzed by immunoblot of SDS total extracts using antibodies against pBAD serine 112. **c** and **d**: Cytoplasmic (CF) and mitochondrial fractions (MF) were obtained from LNCaP cells treated with (**c**) GT3 or (**d**) AT as described above, and analyzed for the presence of cytochrome C by immunoblot. COX IV was used as a mitochondrial marker. **e** and **f**: PC3 cells were grown on 6-well plates and dosed as described above with (**e**) GT3 or (**f**) AT. The SDS total extracts were analyzed for the presence of pBAD serine 112 via immunoblot. **g** and **h**: PC3 cells dosed with (**g**) GT3 or (**h**) AT as described above were processed to obtain cytoplasmic (CF) and mitochondrial fractions (MF) and probed for the presence of cytochrome C and mitochondrial marker COX IV via immunoblot. The values of each analyzed protein were normalized to β-actin. The values are averages ± standard deviations from at least three independent experiments,*p < 0.05. Autoradiographs of the original blots are available in the Supplementary Section Fig. S8.

*There is no significant change in the activated form of BAD in LNCaP and PC3 cells dosed with GT3 or AT.* The loss of phosphorylation of BAD allows it to bind BCL-X_L_ and BCL2 and exert its apoptotic effect [11]. There are two phosphorylation sites on BAD, serine 136 and serine 112. It has been reported that the phosphorylation of serine 136 is dependent on phospho-AKT [12]. We have shown above that there is no change in this protein, and we were unable to detect phospho BAD serine 136 under the described experimental conditions (results not shown). The phosphorylation of BAD at serine 112 requires the activation of the RAF/RAS/ERK pathway [13] and changes may be detectable if the targeted pathway includes mitochondrial involvement. LNCaP and PC3 cells dosed with GT3 show no significant change on the expression levels of phospho-BAD at serine 112 (Fig. 5a and e). When LnCaP and PC3 cells are dosed with AT, a downward trend in the expression levels of this activated form is observed. Statistical analysis of at least three assays show significant decrease (≈56 and 38%) for PC3 when the cells are dosed with 60 and 80 μM of AT, respectively (Fig. 5f, but not for LNCaP cells (Fig. 5b).

*There is no evidence of release of cytochrome c into the cytoplasm in LNCaP and PC3 cells dosed with GT3 or AT.* To test whether the intrinsic pathway of apoptosis is activated by GT3 or AT, we analyzed the levels of cytochrome c in the cytoplasm and the mitochondrial fraction after dosing LNCaP and PC3 and found no significant change as compared to the control for either GT3 (Fig. 5c and g) or AT (Fig. 5d and h). We used COX IV as a mitochondrial marker to confirm the validity of the fractions obtained.

**Fig. 6:**
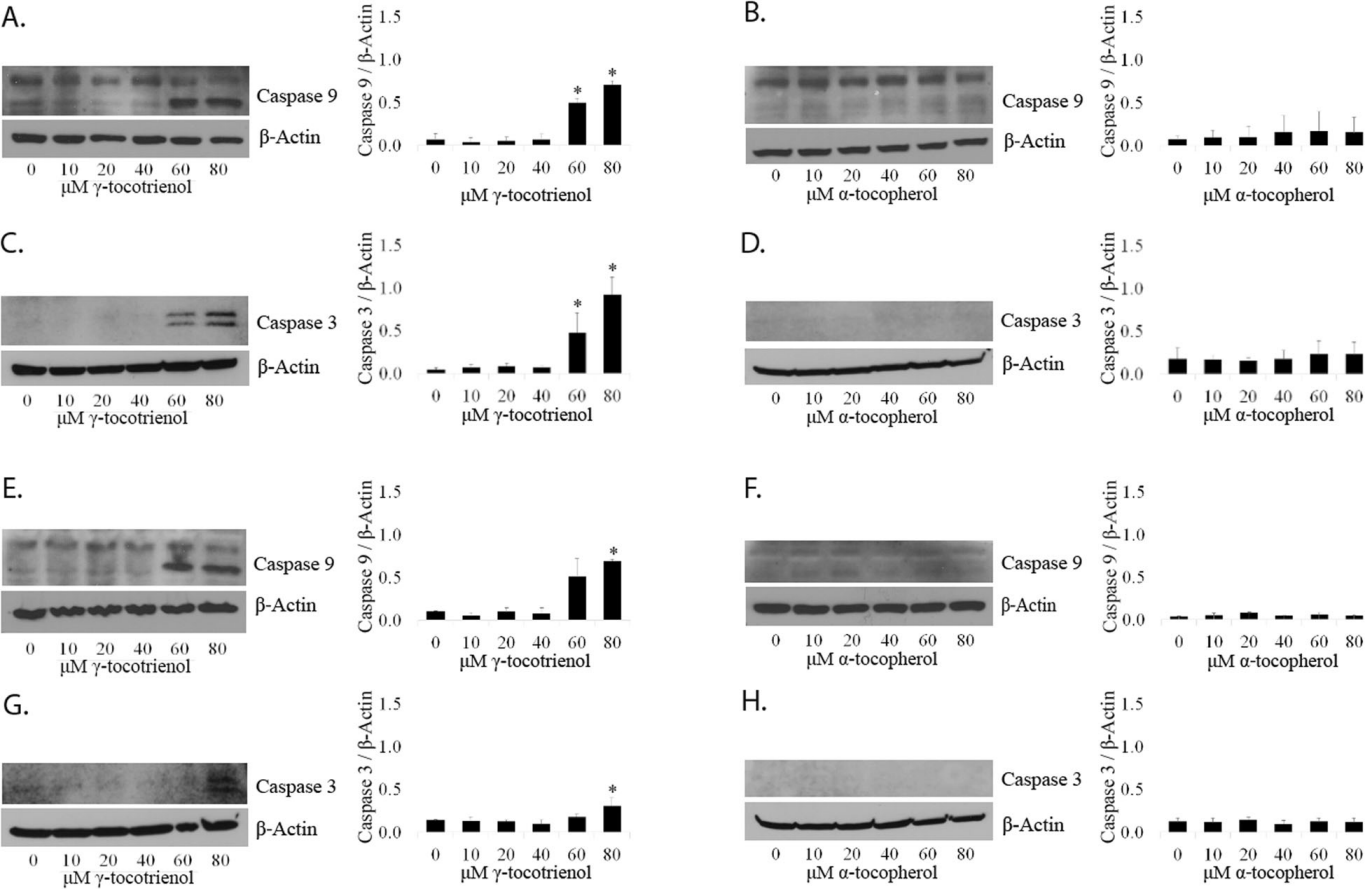
Apoptotic effect of GT3 and AT on prostate cancer cells. **a**, **b**, **c**, and **d**: SDS total extracts of LNCaP cells treated with (**a** and **c**) GT3 or (**b** and **d**) AT at doses ranging from 10 to 80 μM were by immunoblot analyzed for the presence of cleaved caspase 9 (**a** and **b**) and caspase 3 (**c** and **d**). **e**, **f**, **g**, and **h**: In the same manner as described above, PC3 cells treated with (**e** and **g**) GT3 or (**f** and **h**) AT, were processed for immunoblot analysis with antibodies against cleaved caspases 9 and 3. The values of each analyzed protein were normalized to β-actin. The values are averages ± standard deviations from at least three independent experiments; *p < 0.05. Autoradiographs of the original blots are available in the Supplementary Section Fig. S9

*GT3 activates caspases 9 and 3 in LNCaP and PC3 cells but not AT.* The MTT and metabolic assays above had shown the inhibition of cell viability of LNCaP and PC3 cells by GT3. To test whether caspases 9 and 3 were involved in this process, we dosed the cells with GT3 or AT at the indicated concentrations. GT3 caused cleavage of caspases 9 above concentrations of 60 μM in both LNCaP and PC3 cells (Fig. 6a and e). Caspase 3 activation is observed above 60 and 80 μM in LNCaP and PC3 cells respectively (Fig. 6c and g). Conversely, AT causes no detectable effects on caspase 9 after dosing LNCaP and PC3 cells as shown in Fig. 6b and f. Similarly, cleaved caspase 3 is undetectable in these cells when dosed with AT (Fig. 6d and h). The observed results are in agreement with caspase activation by GT3 in prostate cancer cells reported previously [9].

**Fig. 7:**
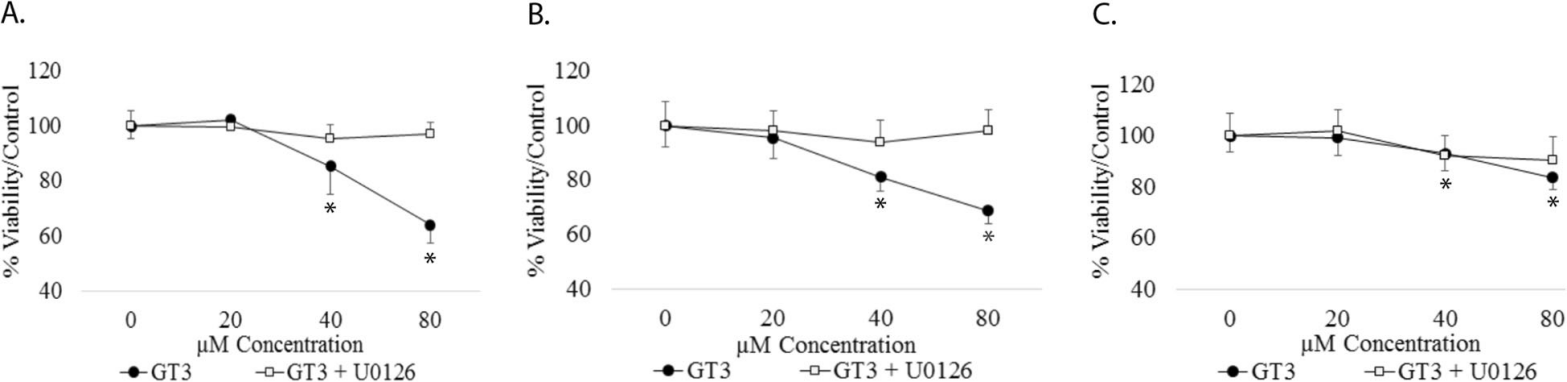
Effect of GT3 and AT on prostate cancer cells in the presence of inhibitor U0126*.***a**, **b** and **c**: Prostate cancer cells (**a**) LNCaP, **b** PC-3, and (**c**) non-tumorigenic RWPE-1 cells were grown in 96-well plates. Two plates were used per cell line; plate one was pre-treated with inhibitor U0126 for 1 h, after which, plates one and two were dosed for 24 h with GT3 at 0, 20, 40 and 80 μM. Cell viability was analyzed by MTT or metabolic assays. For quantification spectrophotometric data were calculated as percentages of the value for the untreated cells (100%) ± standard deviations from at least three independent experiments, *p < 0.05, ***p < 0.001. Statistical analysis using ANOVA and Bonferroni test indicates a significant difference between cells treated with and without inhibitor

*The activation of the RAF/RAS/ERK pathway is essential for the inhibition of cell viability of prostate cells by GT3.* To determine whether the observed activation of the ERK MAPK pathway is relevant to the effect on cell viability of prostate cancer cells by GT3, we used the MEK inhibitor U0126. As shown on Fig. 7a and b, treating LNCaP and PC3 cells with U0126 completely precludes the cytotoxic effect of GT3, suggesting that the phosphorylation of ERK is required for causing apoptosis in these cells. Non-cancerous prostate RWPE-1 cells treated with inhibitor U0126 and GT3 show no difference to cells dosed with GT3 alone (Fig. 7c).

The effect of the inhibitor U0126 on ERK phosphorylation was confirmed by immunoblot of LNCaP cells treated as described. As expected, the co-treatment of the inhibitor and GT3 show also inhibition of the phosphorylation of ERK (Figure S4, supplemental data).

## Discussion

In vitro studies have shown that inhibition of cell viability of prostate cancer cells by GT3 is the result of a multipronged effect that involves the activation of the JNK pathway and suppression of NF-κB via downregulation of EGFR and Id-1. This causes the activation of caspases 9, 8, 7, 3 and PARP cleavage [9]. Activated c-JUN at serine 73 is one of the components of transcription factor AP-1, involved in processes conducive to oncogenesis and cancer progression [14] as well as the initiation of apoptosis. The type of effect observed is cell dependent and is dictated by the characteristics of the molecules activating AP-1. Apoptosis can be initiated via the activation of both, the JNK and ERK pathways, with the subsequent formation AP-1 and caspase cleavage [15]. It has been reported that GT3 treatment of prostate cancer LNCaP and PC3 cells causes upregulation of activated JNK and its target c-JUN via the downregulation of Id-3 and upregulation of MKK4. However, the use of a SP600125, a specific JNK inhibitor, causes a partial block of the downstream phosphorylation of c-JUN, suggesting an additional source of activation. Here we have shown a significant upregulation of phospho-ERK, reported to be required for triggering apoptosis, thus circumventing mechanisms of resistance associated with constitutive activation of MEK1 [16]. Apoptotic effects involving the activation of both, c-JUN and ERK have been previously observed in LNCaP and PC3 prostate cancer cells treated with isothiocyanates, compounds present in cruciferous vegetables [15]. Our studies with inhibitor U0126, suggest that the phosphorylation of ERK is essential for the activation of caspases 9 and 3 and subsequent apoptosis of LNCaP and PC3 cells treated with GT3. However, apoptosis also requires the presence of activated c-JUN as demonstrated by the effect of AT on these cells, where the sole presence of upregulated phospho-ERK will not initiate apoptosis. Thus, the inhibition of cell viability by GT3 in prostate cancer cells requires the presence of the phosphorylated forms of ERK and c-JUN. Our results also exclude the involvement of the intrinsic pathway of apoptosis and the subsequent release of cytochrome c from the mitochondria. This process is initiated by the loss of phosphorylation of BAD at serine 112 or 136. Phospho-ERK is responsible for the activation of BAD at serine 112, whereas activated AKT at serine 473 is responsible for the activation of serine 136. We observed no change in phospho-AKT at serine 473, and were unable to detect phosphorylated BAD at serine 136. Additionally, there were no changes on the levels of activated BAD at serine 112 or on the levels of cytochrome c in the mitochondria. The concentrations of vitamin E isoforms used in this study are in agreement with numerous previous studies and are based on pharmacokinetic reports. Previously determined levels show that circulating vitamin E concentrations range from 3 to 20 μM giving credence to our treatment concentrations as physiologically relevant [17–19].

## Conclusions

The significant upregulation of activated c-JUN in GT3-mediated cell death is consistent with prior studies [9]. However, c-JUN can be phosphorylated by kinases other than JNK, which may allow GT3 to evade prostate cancer cell resistance [20]. Prostate cancer cells treated with GT3 display increased expression of activated ERK which can in turn activate apoptosis regardless of androgen sensitivity. The relevance of the involvement of these two pathways in the apoptotic process has been previously observed before in prostate cancer cells treated with isothiocyanates [15]. Caspase activation by GT3 and subsequent apoptosis is consistent with prior studies [21, 22]. AKT is an important target in prostate cancer therapy and modulates BAD [23, 24], c-JUN [25] and ERK [26]. However, neither the phosphorylation of AKT at serine 473 nor the expression levels of the protein are affected by GT3 under the conditions studied. Sustained activation of the ERK pathway is thought to be one of the mechanisms behind pro-apoptotic effects of cancer drugs [27]. In fact, the phosphorylated forms of ERK and c-JUN can trigger apoptosis without involvement of AKT [15].

## Supplementary information

**Additional file 1.**

**Additional file 2.**

**Additional file 3.**

**Additional file 4.**

**Additional file 5.**

**Additional file 6.**

**Additional file 7.**

**Additional file 8.**

**Additional file 9.**

## Data Availability

All data analyzed during this study are included in this published article and its supplementary information files.

## Abbreviations

GT3: γ-tocotrienol
AT: α-tocopherol
ERK: Extracellular-signal-regulated kinase
JNK: c-Jun N-terminal kinase
SELECT trial: Selenium and vitamin E cancer prevention trial
EGFR: Epidermal growth factor receptor
MTT: (3-[4,5-Dimethylthiazol-2-yl]-2,5-diphenyltetrazolium bromide
PVDF: Polyvinylidene difluoride
BAD: Bcl-2 agonist of cell death
BCL-XL: B-cell lymphoma-extra-large
SDS: Sodium dodecyl sulfate
SDS-PAGE: Sodium dodecyl sulfate–polyacrylamide gel electrophoresis
