# Supplementary figures and images for: Upregulation of pERK and c-JUN by γ-tocotrienol and not α-tocopherol are essential to the differential effect on apoptosis in prostate cancer cells
Source: BMC Cancer. 2020 May 15;20:428. doi: 10.1186/s12885-020-06947-6 (PMC7227069; doi:10.1186/s12885-020-06947-6)

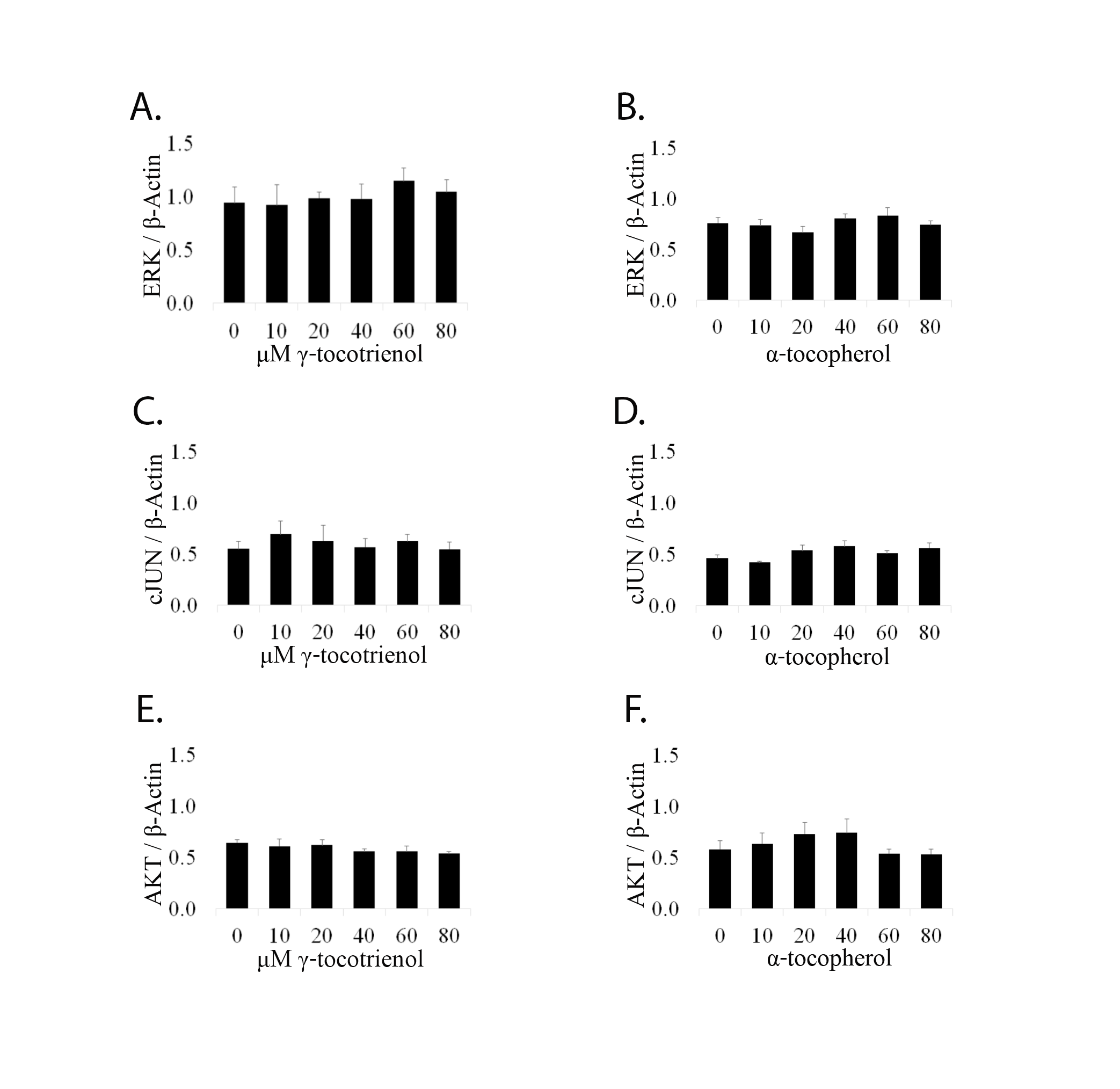

Supplement: Supplementary file 1 — Additional file 1. [file 12885_2020_6947_MOESM1_ESM.tif]

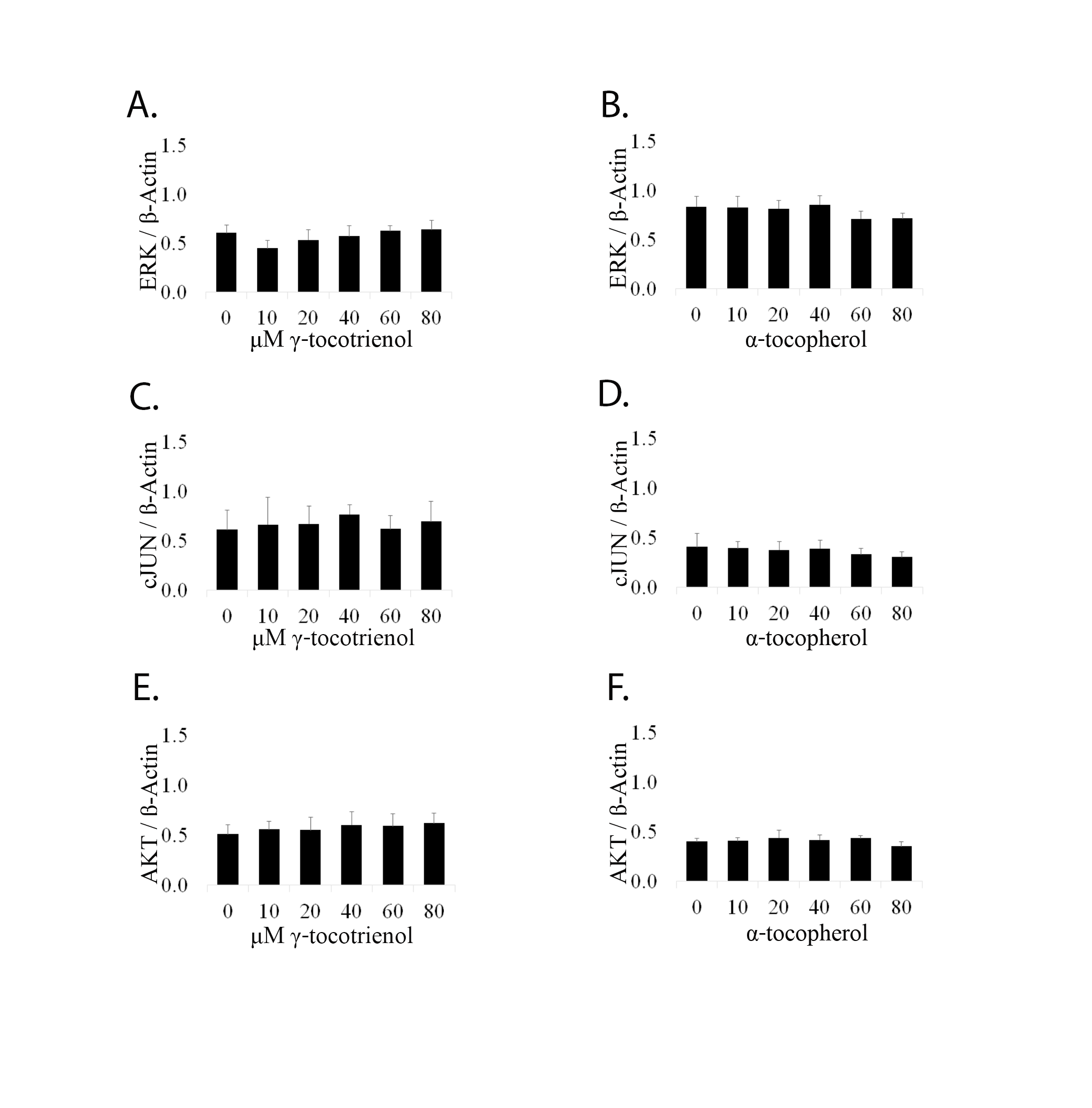

Supplement: Supplementary file 2 — Additional file 2. [file 12885_2020_6947_MOESM2_ESM.tif]

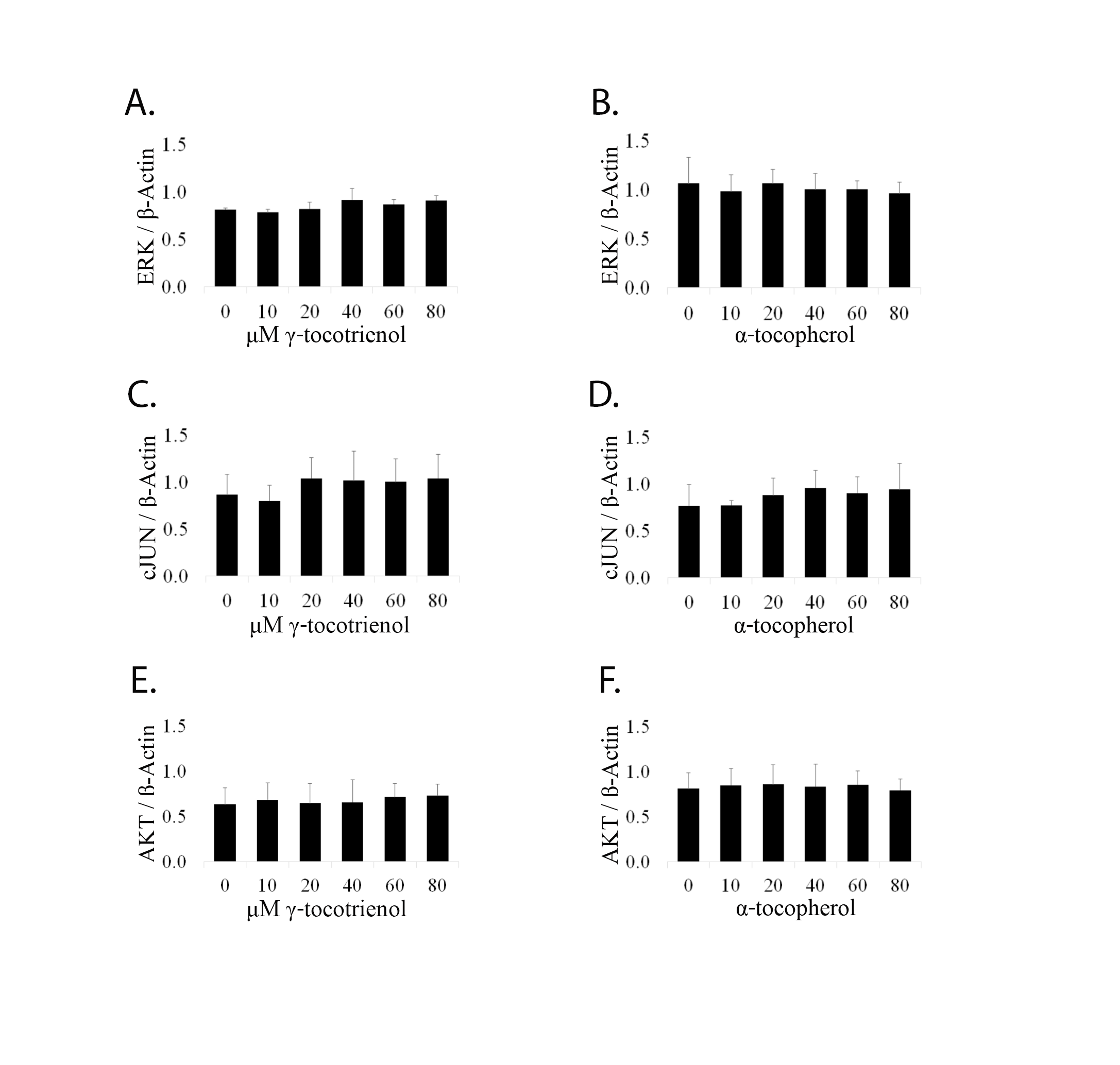

Supplement: Supplementary file 3 — Additional file 3. [file 12885_2020_6947_MOESM3_ESM.tif]

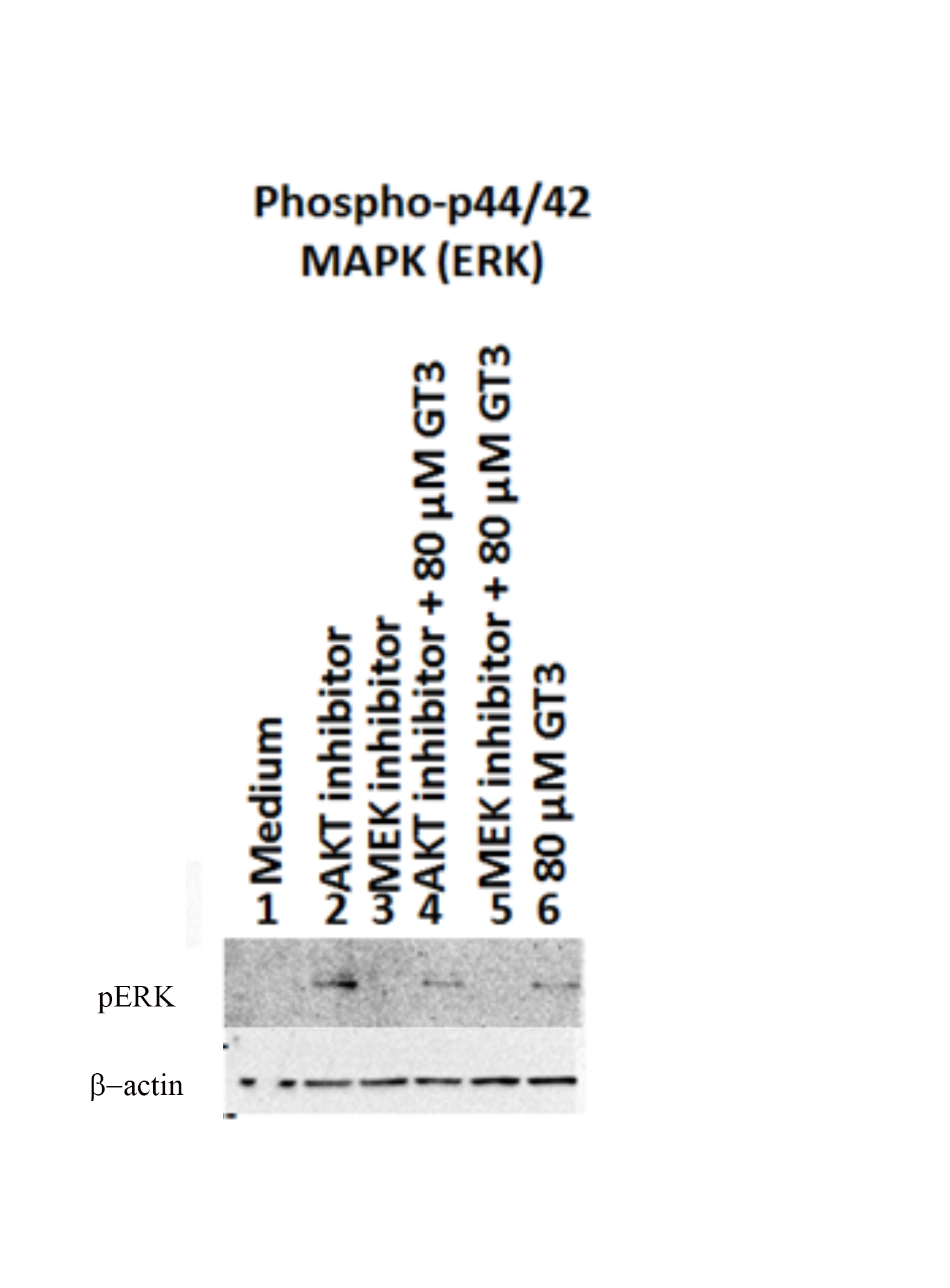

Supplement: Supplementary file 4 — Additional file 4. [file 12885_2020_6947_MOESM4_ESM.tif]

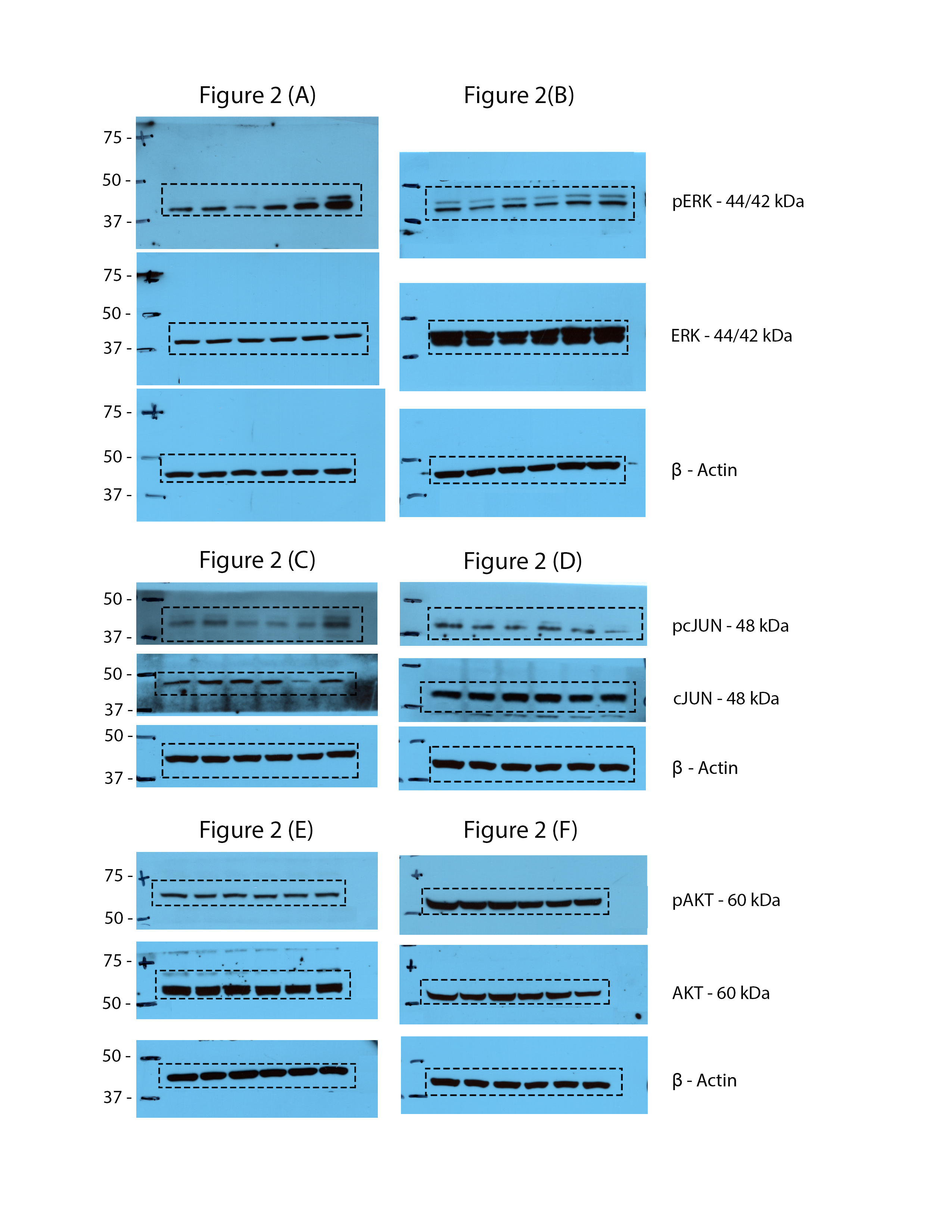

Supplement: Supplementary file 5 — Additional file 5. [file 12885_2020_6947_MOESM5_ESM.tif]

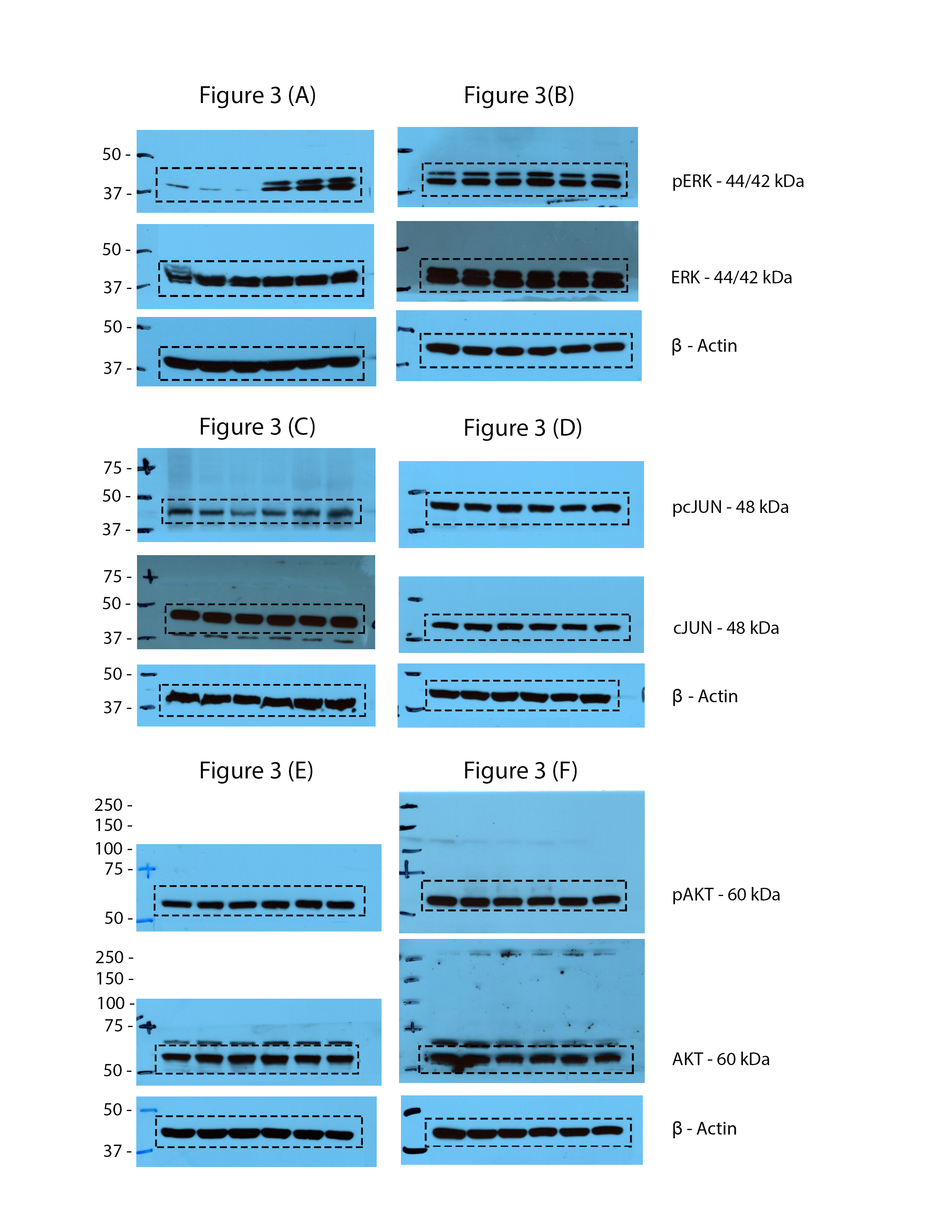

Supplement: Supplementary file 6 — Additional file 6. [file 12885_2020_6947_MOESM6_ESM.tif]

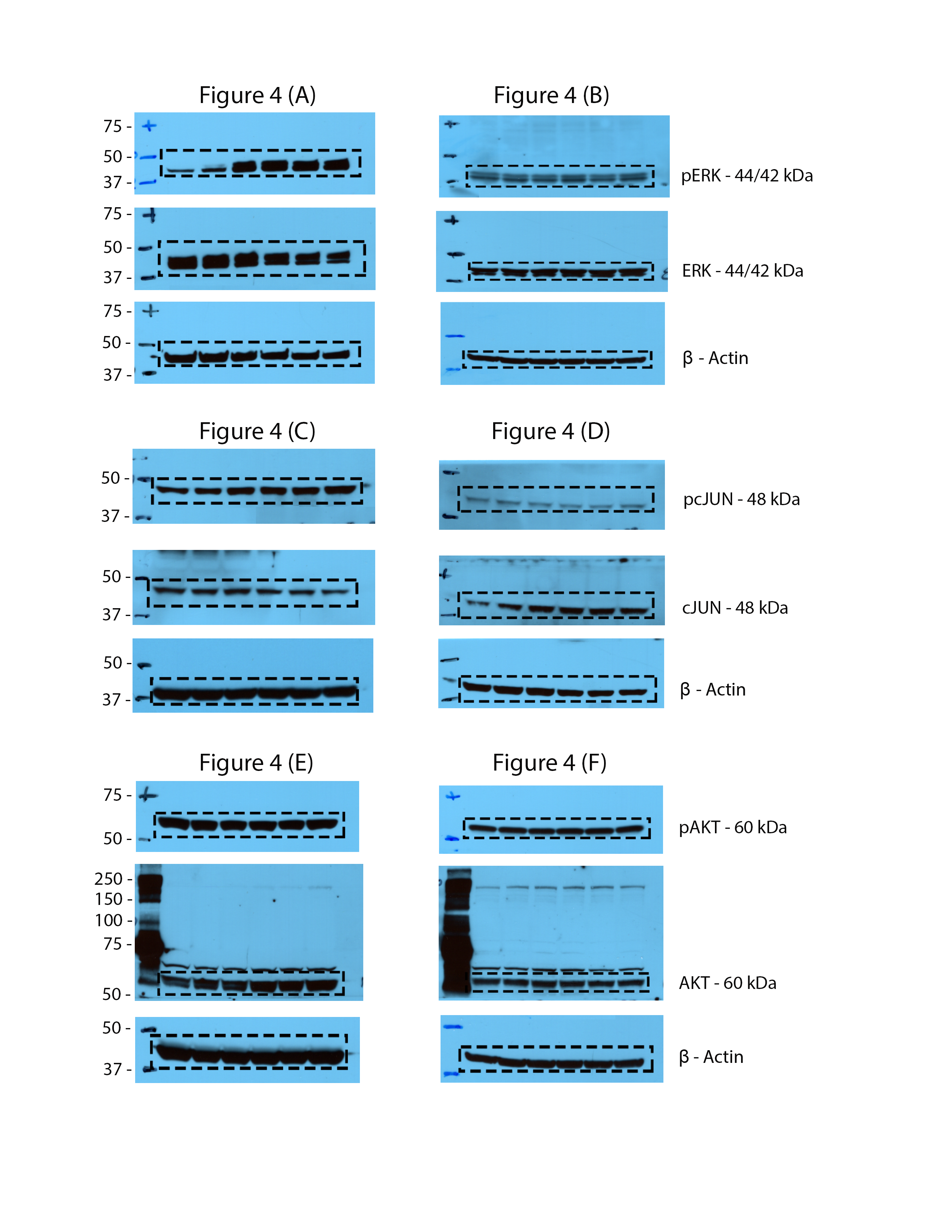

Supplement: Supplementary file 7 — Additional file 7. [file 12885_2020_6947_MOESM7_ESM.tif]

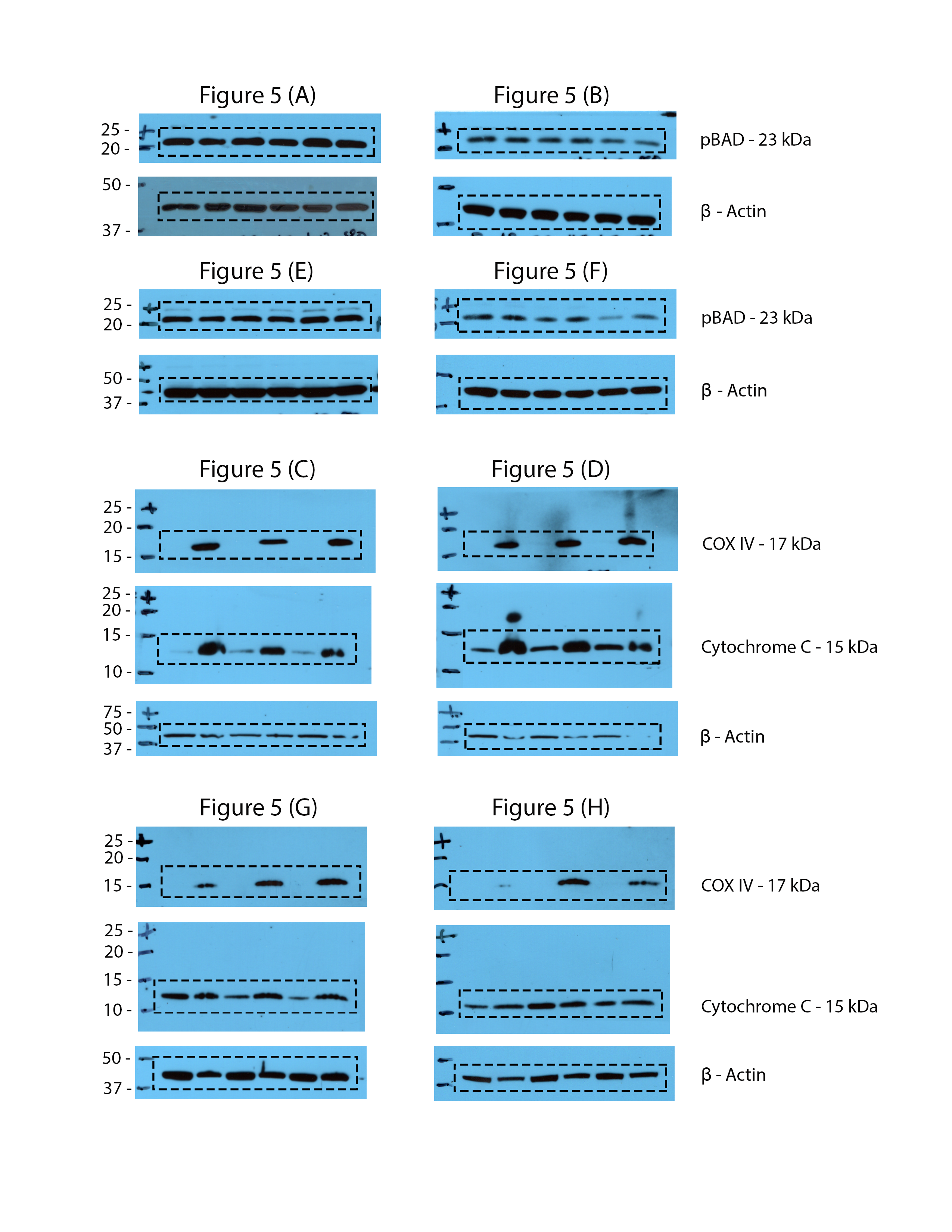

Supplement: Supplementary file 8 — Additional file 8. [file 12885_2020_6947_MOESM8_ESM.tif]

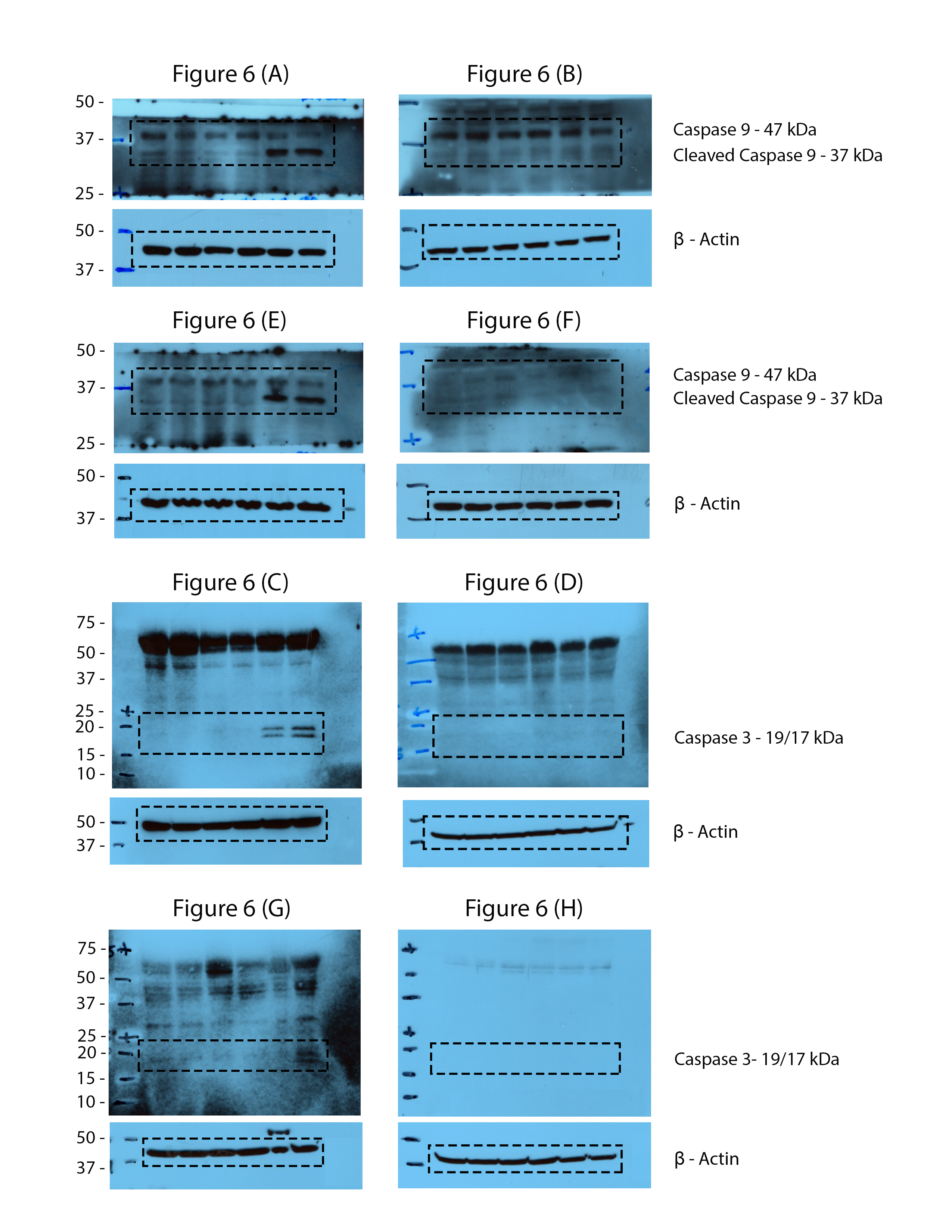

Supplement: Supplementary file 9 — Additional file 9. [file 12885_2020_6947_MOESM9_ESM.tif]
